# Optimal N management affects the fate of urea-^15^N and improves N uptake and utilization of wheat in different rotation systems

**DOI:** 10.3389/fpls.2024.1438215

**Published:** 2024-07-25

**Authors:** Quan Ma, Dandan Dai, Yifan Cao, Qiaoqiao Yu, Xiyang Cheng, Min Zhu, Jinfeng Ding, Chunyan Li, Wenshan Guo, Guisheng Zhou, Xinkai Zhu

**Affiliations:** ^1^ Jiangsu Key Laboratory of Crop Genetics and Physiology, Agricultural College of Yangzhou University, Yangzhou, China; ^2^ Co-Innovation Center for Modern Production Technology of Grain Crops, Yangzhou University, Yangzhou, China; ^3^ Joint International Research Laboratory of Agriculture and Agri-Product Safety, The Ministry of Education of China, Yangzhou University, Yangzhou, China

**Keywords:** ^15^N-labeled urea, wheat, N uptake and utilization, preceding crop, grain yield

## Abstract

Rice-wheat and maize-wheat rotations are major cropping systems in the middle and lower reaches of Yangtze River in China, where high nitrogen (N) inputs and low N efficiency often exacerbate resource waste and environmental pollution. Due to the changes in factors such as soil properties and moisture content, the N fate and the N utilization characteristics of wheat in different rotations are significantly different. Efficient N management strategies are thus urgently required for promoting maximum wheat yield in different rotation systems while reducing N loss. A 2-year field experiment using isotopic (^15^N) tracer technique was conducted to evaluate the fate of ^15^N-labeled urea in wheat fields and the distribution characteristics of N derived from different sources. The wheat yield and N use efficiency under various N rates (180 and 240 kg ha^−1^, abbreviated as N180 and N240) and preceding crops (rice and maize, abbreviated as R-wheat and M-wheat) were also investigated. The results showed that N240 increased N uptake and grain yield by only 8.77−14.97% and 2.51−4.49% compared with N 180, but decreased N agronomic efficiency (NAE) and N physiological efficiency (NPE) by 14.78−18.79% and 14.06−31.35%. N240 also decreased N recovery in plants by 2.8% on average compared with N180, and increased N residue in soil and N loss to the environment. Compared with that of basal N, the higher proportion of topdressing N was absorbed by wheat rather than lost to the environment. In addition, the accumulation of topdressing N in grain was much higher than that of basal N. Compared with that in R-wheat treatment, plants in M-wheat treatment trended to absorb more ^15^N and reduce unaccounted N loss, resulting in higher yield potential. Moreover, the M-wheat treatment increased N recovery in 0−20 cm soil but decreased 80−100 cm soil compared with R-wheat treatment, indicating a lower risk of N loss in deeper soil. Collectively, reducing N application rate and increasing the topdressing ratio is an effective way to balance sustainable crop yield for a secure food supply and environmental benefit, which is more urgent in rice-wheat rotation.

## 1 Introduction

The middle and lower reaches of Yangtze River are one of the major agro-ecosystem regions in China, with winter wheat (*Triticum aestivum* L.)- summer rice (*Oryza sativa* L.) or summer maize (*Zea mays* L.) rotations as the dominant cropping systems. While highly dependent on precipitation irrigation, high inputs of nitrogen (N) fertilizer and high output of food are the characteristics of wheat cultivation in the region. With the increase in global population density and further scarcity of natural resources, the demand for food is steadily increasing. The steady increase in wheat yield, which highly relies on N fertilizer input, has gradually become an important guarantee for solving food security issues (Wu and Ma, 2015; Tan et al., 2021). However, the benefits of N fertilizer in terms of wheat yield are often overestimated, resulting in its overuse (Liu et al., 2017; Wan et al., 2021a). Recent studies have indicated that excessive N inputs and inefficient application practices not only failed to significantly increase crop yields, but also lead to low N use efficiency and high N losses (Chen et al., 2016a; Liu et al., 2018). The above situation is particularly common in China, especially in the middle and lower reaches of Yangtze River, where the N application rates of winter wheat can reach 300 kg ha^−1^ or above (Cui et al., 2014), with an average N recovery efficiency (NRE) of only 20–30% (Ju et al., 2007; Wu and Ma, 2015). A statistical analysis showed that the average N demand of wheat in the middle and lower reaches of Yangtze River was 174 kg ha^−1^, which was beneficial to coordinate yield and N use efficiency (Xu et al., 2021a). The large quantity of unaccountable N inputs can not only delay grain ripening, increase the sensitivity of crops to diseases and pest, and reduce the yield and quality (Zhang et al., 2020; Jug et al., 2021), but also reduce farmers’ profitability and exacerbate the environmental burden (Liu et al., 2018; Guo et al., 2021; Wan et al., 2021b). With the increasingly serious N-related pollution occurring in the middle and lower reaches of Yangtze River, efficient N management practices are of great significance to promote N use efficiency and reduce N loss.

In different crop rotation systems, appropriate N application rate and timing are fundamental to promote the synchronization of soil N supply and crop N uptake, which can ensure crop yield and improve N use efficiency (Fuertes-Mendizábal et al., 2018; Degaspari et al., 2020; Wan et al., 2021a). Generally, there are two peaks of N demand for winter wheat in the middle and lower reaches of Yangtze River, that is, at the seedling stage and after jointing, and the N demand in the later stage is much higher than that in the seedling stage. Wheat growth slows down significantly during winter, when the plants are young and have low N absorption capacity (Ma et al., 2021). During the growing period of wheat, appropriate N supply could enhance the photosynthetic physiological potential of wheat by improving the synthesis of chlorophyll in leaves, which was conducive to the accumulation of assimilates and nutrients in crops (Falcinelli et al., 2022; Sharma et al., 2022). However, excessive N input may shift the balance between vegetative and reproductive growth toward excessive vegetative development, resulting in delayed crop maturation and reduced grain yield, as well as a large amount of N residue in the soil (Wu and Ma, 2015; Liu et al., 2018; Guo et al., 2021). Meanwhile, most of the surplus N escapes from farmlands through nitrate leaching, ammonia volatilization and nitrous oxide emissions (Anas et al., 2020; Wan et al., 2021a), which could damage biodiversity, impair surface and groundwater quality, intensify soil acidification, and aggravate greenhouse gas emissions (Pareja-Sánchez et al., 2020; Zhou et al., 2020; Jia et al., 2023). A previous study in the Taihu region (where has periodic waterlogging in upland systems) has demonstrated that nitrate accumulation and gradual leaching below the root zone were the dominant pathways of N loss in the maize-wheat rotation, which could increase significantly when N supply (550–600 kg ha^−1^) exceeded the crop assimilation capacity (Ju et al., 2009). In the rice-wheat rotation, the unique flood irrigation of rice not only leads to nitrate leaching into deep soil, but also induces a large amount of N loss through runoff (Zhou et al., 2017). In addition, the normal process of gaseous exchange between the soil and the atmosphere is interrupted in flooded soils, which could increase the production of N-containing gases such as nitrous oxide in nitrification–denitrification processes (Tan et al., 2012). Therefore, N inputs should meet but not exceed crop demand, which is crucial to achieve a balance between low N loss and high yield.

The N fate in cropland was also regulated by fertilization timing and the ratio of basal N to topdressing N. Corbeels et al. (1999) believed that crops tend to absorb more N from fertilizer applied earlier rather than later in the growing season. Therefore, traditional fertilization strategies usually involve more N in the early stages of wheat. However, due to the slow expansion of shoots caused by low temperatures in winter, wheat grows slowly for a long time after emergence. This results in a large portion of the basal N not being used in a timely manner, inducing more residual N to migrate downward and leach from the soil profile (Ehdaie et al., 2010; Ma et al., 2021). It has been reported that nitrate leaching can be alleviated by changing the ratio of basal N to topdressing N (Bai et al., 2020; Leng et al., 2021). Previous studies also showed that increasing the proportion of topdressing N was beneficial for improving yield, NRE and crop N uptake (Liu et al., 2019; Cai et al., 2023). Zhang et al. (2023) reported that when the N application rate was 120−240 kg ha^−1^, the optimal ratio of basal N to topdressing N was 6:4, which achieved the highest wheat yield and lowest N loss. While Shi et al. (2012) believed that the optimal proportion of topdressing N accounted for 50%−60% of the total N input. Anyway, it should be clarified that a further increase in crop yield must be achieved via improving N management strategies to increase N use efficiency instead of increasing N application rates.

For improving N use efficiency, the first step is to understand the fate of fertilizer N and the distribution of different sources of N in crops. The use of the isotopic (^15^N) tracer technique has been considered to be an effective approach to achieving this (Shi et al., 2012; Fuertes-Mendizábal et al., 2018), which was an interesting tool for identifying the sources of crop uptake N and quantifying the loss of fertilizer N (Xu et al., 2021b). A previous ^15^N research reported that only 36.6%−38.4% of fertilizer N could be recovered by crops in the current season, while the residual N in soil and unaccounted loss N reached 29.2%−33.6% and 29.5%−34.2%, respectively (Dang et al., 2006). Thanks to the ^15^N tracer technique, scientists were able to explore the fate of fertilizer N, and discover that different previous crops affected the absorption and utilization of fertilizer N by wheat. Wang et al. (2011) showed that in wheat-maize rotation, 69.5%−84.5% of N absorbed by wheat derived from soil, while 6.0%−12.5% and 9.2%−18.1% derived from basal ^15^N and topdressing ^15^N fertilizer, respectively. While in the rice-wheat rotation system, only 43.28%−45.70% of N accumulation in wheat was derived from soil, while 30.11%−41.73% and 13.82%−24.19% were derived from fertilizer N and straw residual N, respectively (Jia et al., 2023).

The different results could be due to the significant differences in soil properties caused by the different growth environments of previous crops, which in turn affected the soil N supply and N loss (Rahimizadeh et al., 2010; Smagacz et al., 2016). However, detailed information on the sources of N uptake by wheat and the fate of fertilizer N as affected by different N management practices in rice-wheat and maize-wheat rotations is still unclear. A better understanding of the fertilizer N fate with ^15^N stable isotope is critical for designing the appropriate N fertilizer programs. Therefore, a 2-year field experiment using ^15^N-labeled urea was conducted to (1) determine the fate of ^15^N-labeled fertilizer in terms of wheat plant accumulation, soil residue and loss to the ecosystem under different N management practices and preceding crops, and assess the characteristics of fertilizer N uptake and utilization by wheat; (2) quantify the distribution of N derived from different sources in different wheat organs, and explore the effective ways to improve N utilization and recovery while ensuring wheat yield under rice-wheat and maize-wheat rotations.

## 2 Material and methods

### 2.1 Experimental site and materials

Field experiment was carried out in 2013−2015 at the Agricultural Experiment Station of Agricultural College (32°39′ N, 119°42′ E), Yangzhou University, China. The experiment site is located in the middle and lower reaches of the Yangtze River with a typical subtropical monsoon climate. The accumulated temperature, precipitation and sunshine duration during the wheat growing seasons in 2013−2015 were shown in **Table 1**. The soil type was silt loam, and the primary properties of the 0−100 cm soil layers were determined before sowing (**Table 2**).

**Table 1:** Climatic conditions during the wheat-growing season at the experimental site in 2013−2015.

Stage	Accumulated temperature (°C)	Accumulated precipitation (mm)	Accumulated sunshine duration (h)
2013−2014			
SD−OW	629	33	348
OW−JS	361	148	359
JS−BS	593	182	232
BS−MS	870	88	281
WGP	2453	451	1220
2014−2015			
SD−OW	612	104	310
OW−JS	385	82	354
JS−BS	518	140	209
BS−MS	844	136	266
WGP	2359	462	1139

SD, Sowing date; OW, Over-wintering stage; JS, Jointing stage; BS, Booting stage, MS, Maturity stage; WGP, Whole growth period.

**Table 2:** Primary properties of soil (0−100cm) pre-sowing in the experimental field.

Preceding crop	Soil layer (cm)	Organic matter (g kg^−1^)	Total N (g kg^−1^)	Available N (mg kg^−1^)	Available P (mg kg^−1^)	Available K (mg kg^−1^)
2013−2014						
Rice	0−20	13.36	0.611	57.37	9.27	91.60
	20−40	12.47	0.581	50.43	7.44	85.62
	40−60	10.84	0.436	38.57	7.33	89.74
	60−80	11.25	0.439	39.93	8.13	77.68
	80−100	8.39	0.385	36.44	5.70	80.24
2014−2015						
Rice	0−20	14.43	0.589	57.13	11.45	89.31
	20−40	11.57	0.557	50.58	10.70	81.63
	40−60	11.40	0.476	39.37	8.22	80.59
	60−80	12.64	0.475	40.43	8.46	73.26
	80−100	8.86	0.414	39.93	7.53	74.86
Maize	0−20	13.52	0.619	63.03	10.18	91.86
	20−40	11.25	0.580	55.87	9.51	79.49
	40−60	12.06	0.502	43.52	9.53	80.35
	60−80	11.48	0.528	38.38	7.52	81.75
	80−100	9.15	0.419	38.57	6.69	70.11

The rice and maize varieties were Ningjing 1 and Suyu 20, respectively. In the rice growing season, the N application rate was 180 kg ha^−1^. The rice was sown on 10 May 2013 and 11 May 2014, and harvested on 25 October 2013 and 27 October 2014. The rice seedlings were raised under dry farming conditions, with the row spacing of 20 cm × 20 cm and 2 plants per hole. In the maize growing season, the N application rate was 225 kg ha^−1^, with the density of 6 × 10^4^ plants ha^−1^. The sowing and harvest dates of maize were 29 June 2014 and 15 October 2024, respectively. After rice and maize being harvested, the residues were retained to the filed. The wheat cultivar used in this study was Yangmai 16, which was bred by Lixiahe Institute of Agriculture Sciences, Jiangsu, China. The ^15^N-labeled urea (with 10.18% abundance of ^15^N) was produced by Shanghai Research Institute of Chemical Industry, Shanghai, China. The N, P and K fertilizers used in the experiment were urea (46% N), superphosphate (12% P_2_O_5_) and potassium chloride (60% K_2_O).

### 2.2 Experimental design and field management

In 2013−2014, the experiment was conducted only in the field after rice harvest using a single-factor randomized block design with two N rates of 180 kg ha^−1^ (termed N180) and 240 kg ha^−1^ (termed N240). In 2014−2015, the experiment was laid out using a split-plot design with the preceding crops (rice and maize, abbreviated as R-wheat and M-wheat) as the main plot and N rates (N180 and N240) as the subplot. In each treatment, 50% urea was applied as basal N, and the rest as topdressing N. The topdressing N was divided into three times: 10% applied at the four-leaf stage, 20% applied at the jointing stage and 20% applied at the booting stage. An additional blank control without N application was set up to calculate N efficiency for different treatments. In all treatments, the phosphorus (120 kg ha^−1^ P_2_O_5_) and potassium (120 kg ha^−1^ K_2_O) fertilizers were applied once pre-sowing. The wheat was artificially sown on November 7, 2013 and November 2, 2014, respectively, with the row spacing of 30 cm and depth of 2−3 cm. Each treatment was applied to three plots as replicates and each plot was 2.8 m long × 2.2 m wide. The seedling density was uniformly adjusted to 180 × 10^4^ plants ha^−1^ at the three-leaf stage. Other field practices were consistent with the local high-yield guidelines.

A micro-plot isolated by an iron frame (45 cm long ×15 cm wide × 35 cm high) was set up to monitor the fate of ^15^N-labeled urea in each plot. The iron frame was inserted 30 cm deep into the soil layer with 5 cm exposed to the soil surface to prevent surface runoff and lateral contamination. In the micro-plots, the common urea was replaced with ^15^N-labeled urea at the similar timing and rate as the main plot. To evaluate the effects of different N sources on the fate of fertilizer N, two additional micro-plots were set up in the N240 treatment: ^15^N-labeled urea applied as basal N, common urea applied as topdressing N and common urea applied as basal N, ^15^N-labeled urea applied as topdressing N. Other agronomic practices in the micro-plots followed the relevant main plot.

### 2.3 Sampling and chemical analyses

#### 2.3.1 Yield, yield components and N use efficiency calculation

At the maturity stage, an area of 1.2 m^2^ per plot was randomly selected to count spike number, and determine the grain yield after being harvested, threshed and dried naturally. Three subsamples of 1000 grains were randomly selected from the harvest grains to measure 1000-grain weight. The grain yield and 1000-grain weight were reported at a stand moisture content of 13%. 20 plants per plot were sampled and the number of grains per spike was measured before dried at 80°C in an oven to a constant weight. The plant samples were crushed after weighting. The N concentration of plant samples was determined by the Kjeldahl method (Douglas et al., 1980). The total N accumulation of wheat was the product of plant N concentration and dry matter accumulation. N agronomic efficiency (NAE), N physiological efficiency (NPE) and N harvest index (NHI) were calculated as follows (**Equations 1**–**3**) (Wan et al., 2021b):


(1) $$\text{NAE}({\text{kg kg}}^{-1})=\frac{\text{Grain yield in N treated plot}-\text{Grain yield in zero N plot}}{\text{N rate}}$$

(2) $$\text{NPE}({\text{kg kg}}^{-1})=\frac{\text{Grain yield in N treated plot}-\text{Grain yield in zero N plot}}{\text{N uptake in N treated plot}-\text{N uptake in zero N plot}}$$

(3) $$\text{NHI}=\frac{\text{N uptake by grain}}{\text{N uptake by plant}}$$

#### 2.3.2 Plant and soil sampling in micro-plots and ^15^N analysis

At the maturity stage, all plant samples in the micro-plots were harvested and divided into different organs (leaf, Stem and sheath, grain, Ear axis and glume) before being dried at 80°C in an oven to a constant weight. The plant samples were crushed and passed through a 150 μm sieve for N concentration and ^15^N analysis. After wheat harvest, the soil samples were collected to a depth of 100 cm in 20 cm increments in the micro-plots following the method described by Jayasundara et al. (2010). The soil samples were air-dried and ground to pass through a 150 μm sieve for total N content and ^15^N analysis. Soil total N content was determined using an elemental analyzer (Costech ECS4010, Costech Analytical Technologies Inc., Valencia, USA). The ^15^N concentration of all samples was determined by isotope ratio mass spectrometry (Isoprime 100, Elementar, Britain). All ^15^N was expressed as the atom percent excess corrected for background abundance (i.e. 0.366%). N derived from fertilizer (Ndff) in plants and the soil residual ^15^N during the growing season were calculated according to López-Bellido et al. (2005) (**Equations 4**–**6**):


(4) Ndff in plant(%)=N 15 atom% in plantN 15 atom% in fertilizer

(5) Ndff in soil(%)=N 15 atom% in soilN 15 atom% in fertilizer

(6) Soil residual N 15(kg ha−1)=Soil total N×Ndff in soil

### 2.4 Data processing and statistical analysis

Microsoft Excel 2010 (Microsoft Corporation, Washington, USA) and Origin 95 (OriginLab, USA) were adopted for data processing and figure drawing, respectively. Data was subjected to analysis of variance (ANOVA) for each year using DPS 7.05 (Zhejiang University, Hangzhou, China). In 2013−2014, a one-way analysis of variance (ANOVA) was performed since rice was the only preceding crop. While in 2014−2015, a two-way analysis of variance (ANOVA) was performed to evaluate the effects of preceding crops and N management on the measured parameters. The main effects of preceding crop, N application rate and their interactions were tested for all measured characters. Mean values of the treatments were compared based on the least significant difference test (*P*< 0.05).

## 3 Results

### 3.1 urea-^15^N fate

**Figure 1:**
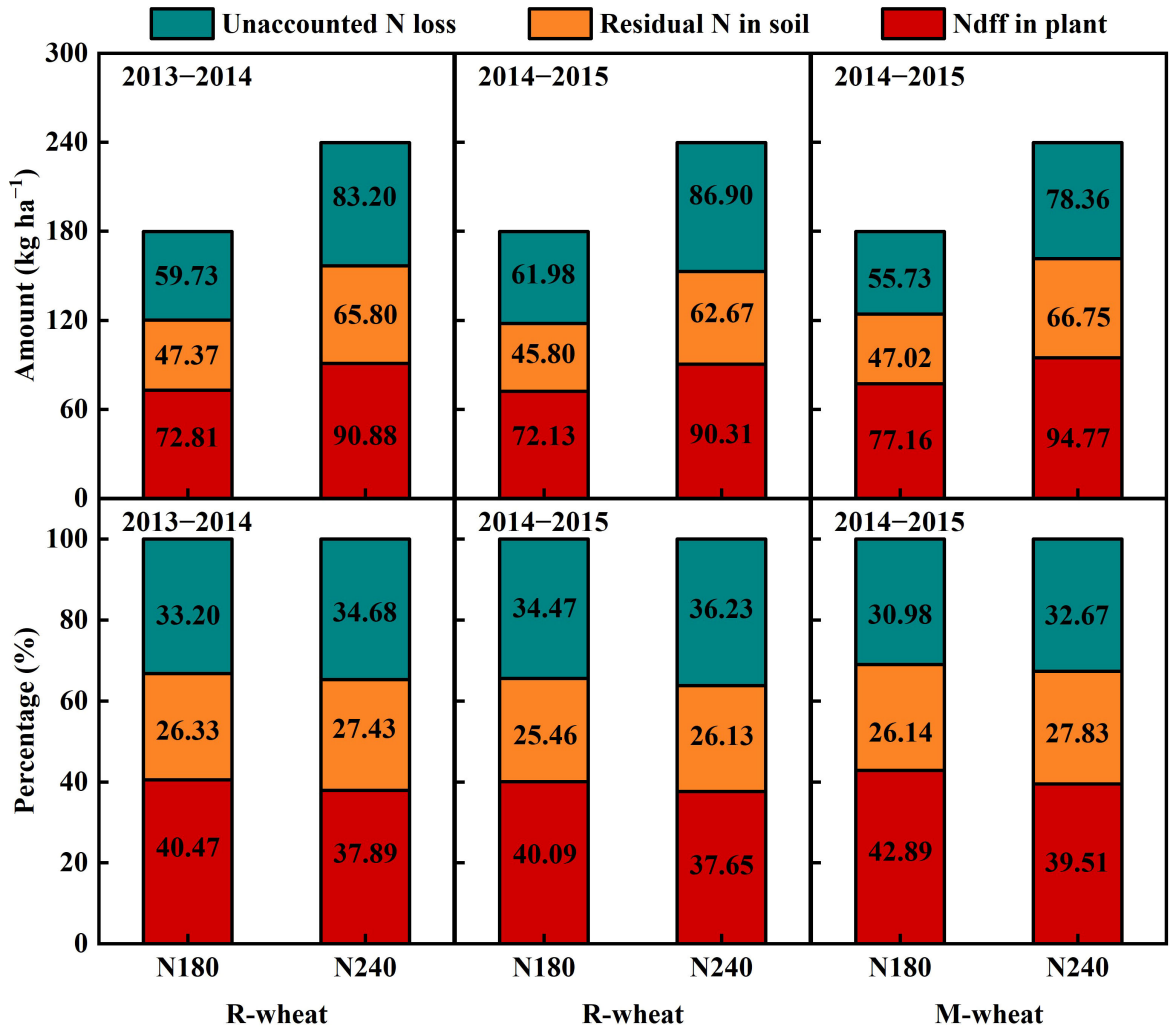
The fate of fertilizer N during the wheat growing season under different preceding crops and N rates in 2013−2015. Ndff, N derived from fertilizer.

In different N rates and preceding crops, the fate of urea-^15^N during the wheat growing season was in the order of Ndff in the plant > unaccounted N loss > residual N in soil (**Figure 1**). When the preceding crop was rice or maize, N180 was observed to decrease the amount of Ndff in plant compared with N240, with an average decrease of 24.28% in 2013−2015. However, N180 increased the percentage of Ndff in plant by 2.8% on average compared with N240. The amount of unaccounted N loss increased with increasing N rate, but the percentage showed an opposite trend compared with Ndff in plant, which decreased by 1.65% in N180 compared with that in N240. With the equal N rate, the preceding maize treatment increased the percentage of Ndff in plant and residual N in soil, but decreased the percentage of unaccounted N loss compared with the preceding rice treatment.

**Figure 2:**
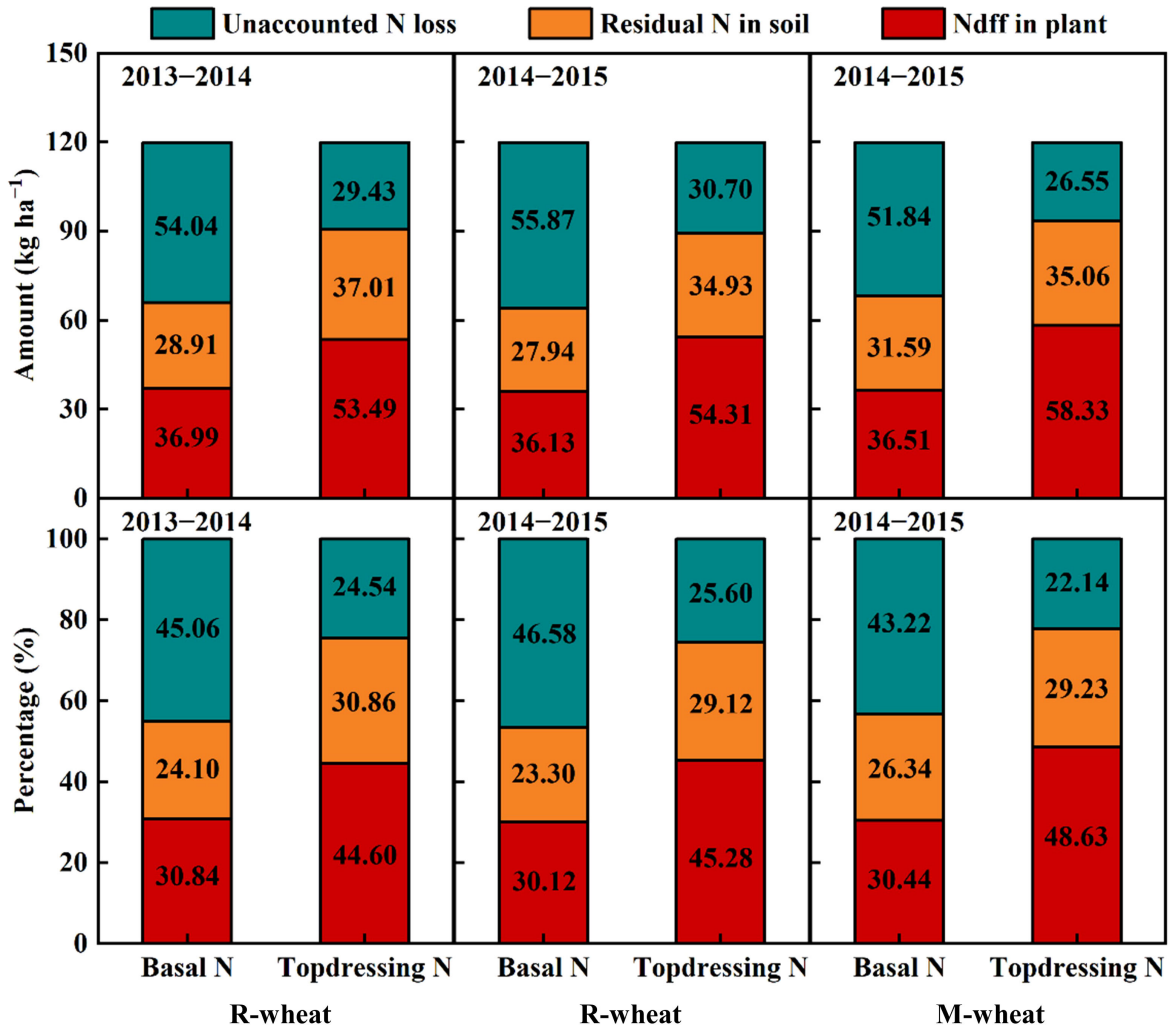
The fate of fertilizer N from different application periods during the wheat growing season in 2013−2015. Ndff, N derived from fertilizer.

The different fertilization periods also greatly affected the fate of urea-^15^N during the wheat growing season (**Figure 2**). When the preceding crop was rice or maize, the amount and percentage of Ndff in plant and residual N in soil from basal N were obviously lower than those from topdressing N, but the opposite trend was observed in the unaccounted N loss. In 2014−2015, the N derived from basal N in plant showed no difference between the preceding rice and maize treatment, while the preceding maize treatment increased the N derived from topdressing N in plant by 3.35% and decreased the unaccounted N loss by 3.46% compared with the preceding rice treatment.

### 3.2 Distribution of residual urea-^15^N in different soil layers

**Figure 3:**
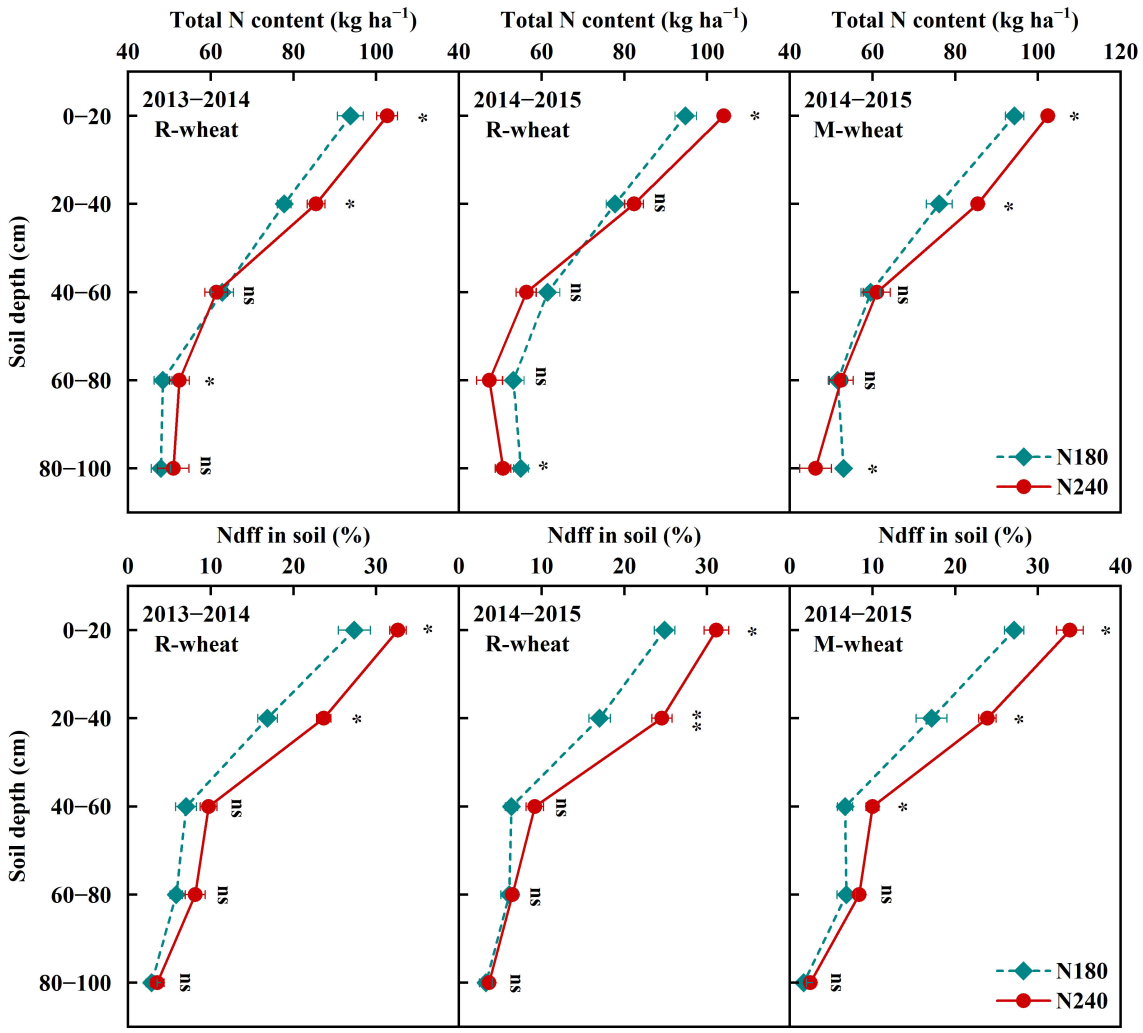
Distribution of the total N content and N derived from fertilizer (Ndff) in soil under different preceding crops and N rates in 2013−2015. * indicates significance at *P* < 0.05; ** indicates significant at P<0.01; ns indicates no significance.

The residue of urea-^15^N in soil decreased gradually with the increase of soil depth, which was consistent with the trend of total N content (**Figure 3**). With the increase of N rate, N240 increased the Ndff in different soil layers compared with N180, with an average increase of 6.09% in 0−20 cm soil depth, and 7.02% in 20−40 cm soil depth. With the same N rate, the preceding maize treatment increased the Ndff by 2.51% on average in 0−20 cm soil layers compared with the preceding rice treatment. When the N rate was 180 kg ha^−1^, the total Ndff of preceding rice and maize treatment in 0−100 cm soil layers was 13.38% and 14.06%, respectively, which was 14.06% and 19.21%, respectively, when the N rate was 240 kg ha^−1^. The results indicated that the preceding maize and higher N application rate contributed to the residue of fertilizer N in soil.

**Figure 4:**
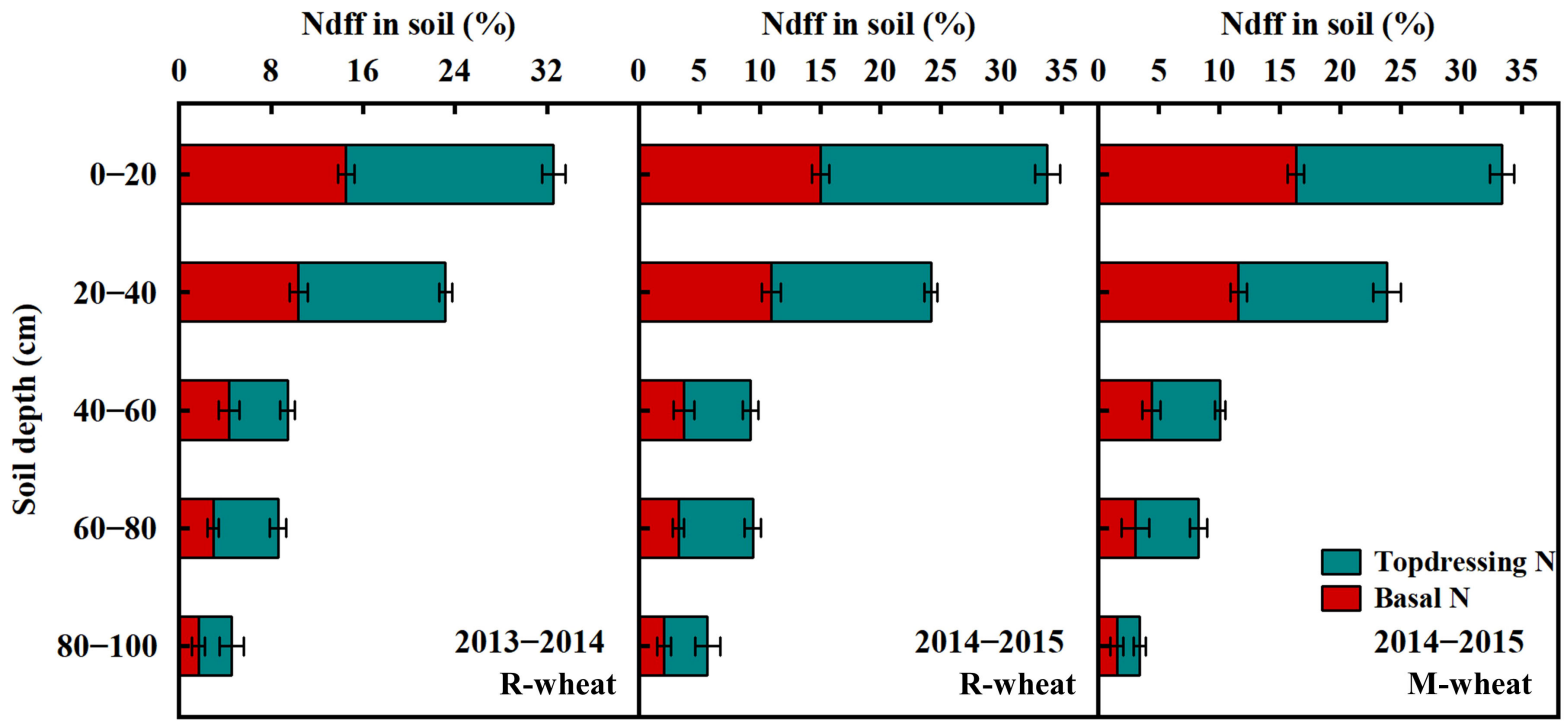
Distribution of the N derived from basal fertilizer and topdressing fertilizer in soil at N rate of 240 kg ha^−1^ in 2013−2015.

Further analysis showed that the residue of urea-^15^N in soil was mainly concentrated in 0−40 cm soil layer (**Figure 4**). In the same soil layer, the residual N in soil from topdressing N was obviously higher than that from basal N. when the preceding crop was rice or maize, no significant difference was observed in the N residue from basal N or topdressing N.

### 3.3 Sources of wheat uptake N and its distribution in different organs

Observations showed that when the preceding crop was rice or maize, the N uptake by wheat at the maturity stage was 55.97%%−62.60% from soil and 37.40%−44.03% from fertilizer in 2013−2015 (**Table 3** and **Supplementary Table 1**). Under the same preceding corp, N240 significantly increased the total N uptake by wheat and the ratio of Ndff in different organs compared with N180, but significantly decreased the ratio of Ndfs in different organs. N distribution in different wheat organs was in the order of grain > stem and sheath > leaf > ear axis and glume. The preceding crop and N rate had different effects on N distribution in different wheat organs (**Supplementary Table 2**). In leaf and stem and sheath, the preceding maize treatment increased the total N uptake, Ndff and Ndfs compared with the preceding rice treatment. In grain, the preceding maize treatment showed a trend of increasing Ndff but decreasing Ndfs compared with the preceding rice treatment.

**Table 3:** Distribution of N derived from fertilizer (Ndff) and from soil (Ndfs) in wheat plants under different preceding crops and N rates in 2014−2015.

Organ	Preceding crop	N rate	Total N uptake (kg ha^−1^)	Ndff		Ndfs	
				Amount (kg ha^−1^)	Ratio (%)	Amount (kg ha^−1^)	Ratio (%)
Leaf	Rice	N180	13.86c	4.89c	35.31b	8.97b	64.69a
		N240	16.60a	6.96a	41.93a	9.64a	58.07b
	Maize	N180	15.00b	5.50b	36.65b	9.50ab	63.35a
		N240	16.99a	7.16a	42.15a	9.83a	57.85b
Stem and sheath	Rice	N180	29.58c	10.19c	34.44b	19.39c	65.56a
		N240	37.38a	15.97a	42.72a	21.41b	57.28b
	Maize	N180	31.96b	11.32b	35.40b	20.65b	64.60a
		N240	39.21a	16.27a	41.49a	22.95a	58.51b
Gram	Rice	N180	134.70b	51.47d	38.21c	83.23a	61.80a
		N240	140.80ab	60.91b	43.26ab	79.88c	56.74bc
	Maize	N180	135.76b	55.55c	40.92b	80.20b	59.08ab
		N240	146.99a	65.45a	44.52a	81.55ab	55.48c
Ear axis and glume	Rice	N180	14.72ab	5.58b	37.91b	9.14a	62.09a
		N240	15.21a	6.47a	42.50a	8.74ab	57.50b
	Maize	N180	13.34c	4.80c	35.95b	8.55bc	64.05a
		N240	13.95bc	5.90b	42.26a	8.05c	57.74b
Total	Rice	N180	192.86b	72.13c	37.40b	120.74a	62.60a
		N240	209.99a	90.31a	43.00a	119.68ab	57.00b
	Maize	N180	196.05b	77.16b	39.36b	118.89b	60.64a
		N240	217.14a	94.77a	43.64a	122.37a	56.36b

When the preceding crop was rice or maize, the ratio of Ndfbf in different wheat organs was obviously lower than that of Ndftf (**Table 4** and **Supplementary Table 3**). The preceding maize crop showed a trend of decreasing the ratio of Ndfbf but increasing the ratio of Ndftf in wheat plants compared with the preceding rice treatment, and similar results were observed in leaf, stem and sheath and ear axis and glume. The preceding maize treatment showed no significant difference in the ratio of Ndfbf, but significantly decreased the ratio of Ndftf compared with the preceding rice treatment.

**Table 4:** Distribution of N derived from basal fertilizer (Ndfbf) and from topdressing fertilizer (Ndftf) in wheat plants at N rate of 240 kg ha^−1^ in 2014−2015.

Organ	Preceding crop	Ndfbf		Ndftf	
		Amount (kg ha^−1^)	Ratio (%)	Amount (kg ha^−1^)	Ratio (%)
Leaf	Rice	2.85a	17.20a	4.12b	24.83b
	Maize	2.77b	16.29b	4.37a	25.72a
Stem and sheath	Rice	6.75a	18.06a	9.04b	24.19b
	Maize	6.49b	16.55b	10.14a	25.85a
Grain	Rice	23.78b	16.89a	37.39b	26.56b
	Maize	24.96a	16.98a	40.10a	27.28a
Ear axis and glume	Rice	2.75a	18.06a	3.75a	24.68b
	Maize	2.29b	16.40b	3.72a	26.67a
Total	Rice	36.13a	17.20a	54.31b	25.86b
	Maize	36.51a	16.81b	58.33a	26.86a

### 3.4 Grain yield and yield components

The preceding crop and N rate significantly affected wheat yield and yield components (**Table 5**). In 2013−2014, there was no significant difference in grain yield between N180 and N240 in the preceding rice treatment. But in 2014−2015, N240 significantly increased grain yield compared with N180 in both preceding rice and maize treatments. Under the same N rate, the preceding maize treatment showed a slightly higher yield potential compared with the preceding rice treatment, but the difference was not significant. Under the same preceding crop, N240 significantly increased spikes compared with N180, but significantly decreased kernels per spike and 1000-grain weight. Compared with rice, the preceding maize treatment showed an obvious trend of increasing spikes and kernels per spike, but decreasing 1000-grain weight under the same N rate.

**Table 5:** Grain yield and yield components of wheat under different preceding crops and N rates in 2013−2015.

Preceding crop	N rate	Spikes (kg ha^−1^)	Kernels per spike	1000-grain weight (g)	Grain yield (kg ha^−1^)
2013−2014					
Rice	N180	387.69b	44.40a	42.03a	6928.87a
	N240	435.22a	42.43b	40.24b	7102.91a
2014–2015					
Rice	N180	386.18d	44.79ab	41.95a	6904.36c
	N240	433.55b	43.09c	40.97c	7144.86ab
Maize	N180	399.06c	45.36a	41.62ab	6945.61bc
	N240	452.88a	43.51b	38.83d	7257.41a

### 3.5 N use efficiency

NAE, NPE and NHI under different preceding crops and N rates were shown in **Table 6**, which indicated the absorption and utilization of N by crops. Under the same preceding crop, N240 significantly decreased NAE and NPE compared with N180. NHI in N240 also showed the trend of lower than that in N180, but the significant difference was observed only in the preceding rice treatment in 2014−2015. With the same N rate, the preceding maize treatment achieved lower NPE compared with the preceding rice treatment.

**Table 6:** N agronomic efficiency (NAE), N physiological efficiency (NPE) and N harvest index (NHI) of wheat under different preceding crops and N rates in 2013−2015.

Preceding crop	N rate	NAE (kg kg^−1^)	NPE (kg kg^−1^)	NHI
2013−2014				
Rice	N180	11.71a	45.81a	0.69a
	N240	9.51b	31.45b	0.67a
2014−2015				
Rice	N180	13.03a	38.70a	0.70a
	N240	10.77b	33.26c	0.67b
Maize	N180	12.79a	36.59b	0.69ab
	N240	10.90b	31.12d	0.68ab

## 4 Discussion

### 4.1 The characteristics of urea-^15^N fate under different N management strategies and preceding crops

In agricultural ecosystems, fertilizer N applied to the soil generally has three fates: uptake by crop, residual in the soil and loss through various pathways. Many studies have shown that the fates of fertilizer N are closely related to N fertilizer amount, fertilization practice, cropping system, soil fertility, climatic factors, etc (Harris et al., 2016; Wan et al., 2021b; Jia et al., 2023). In our study, ^15^N tracer technique was used to analyze the effect of N rate and fertilization timing on the fate of fertilizer N in rice-wheat and maize-wheat systems. Regardless of whether the preceding crop was rice or maize, N240 increased the amount of fertilizer N uptake by wheat, but reduced the N recovery in plants from 41.15% (40.09%−42.89) to 38.35% (37.65%−39.51%) at the maturity stage compared with N180. As Dobermann et al. (2006) found, the major factor determining N recovery efficiency was N rate. A study conducted on winter wheat in a loamy soil confirmed that increasing the N application rate from 60 to 240 kg ha^−1^ reduced the N recovery in plants from 33.05% to 24.85% (Chen et al., 2016b). Although higher N input could increase crop uptake of fertilizer N, it was accompanied by more fertilizer N residue in the soil or loss to the environment. Part of fertilizer N not absorbed by crops in the current year could be retained by the soil and reserved for use by subsequent crops (Chen et al., 2016b). However, excessive N accumulation in soil significantly decreased N use efficiency and increased the risk of nitrate leaching to deep soil (Wang et al., 2011; Jia et al., 2023), especially in rice-wheat rotation systems. In the process of rice planting, soil flooding leads to high soil moisture after rice harvest, which changed the soil physical and chemical structure and microbial community distribution, aggravating nitrate leaching (Jing et al., 2009; Jia et al., 2011). A study in the Middle and Lower Yangtze River Basin in China also showed that frequent precipitation and higher groundwater table could induce residual N leaching into deep soil in the rice-wheat rotation systems (Liang et al., 2011), and high N inputs often exacerbated the above process (Wang et al., 2011). Previous studies have confirmed that the low seasonal rainfall and soil moisture could reduce soil N leaching loss and gaseous N emissions to the atmosphere (Liang et al., 2020). The regression analysis also conducted by Wang et al. (2016a) also showed that cumulative air temperature and precipitation had significant positive influences on cumulative ammonia volatilization losses and denitrification losses. Compared with that in the preceding maize treatment, the higher potential of soil residual N loss in the preceding rice treatment could explain the lower N recovery in plants.

Generally, in addition to high N inputs and superfluous precipitation, an unreasonable ratio of basal N to topdressing N also increased the risk of N loss (Cai et al., 2023). Ye et al. (2022) suggested that the plant uptake and residual of basal N were lower than those of topdressing N in the wheat growing season, whereas N loss showed an opposite trend. In our study, the basal N recovery averaged 30.47% in plants and 24.58% in 0−100 cm soil, respectively, which was obviously lower than topdressing N recovery in plants (46.17%) and soil (29.74%). In contrast, basal N resulted in significantly higher N loss compared with topdressing N. This finding was consistent with Wang et al. (2011), who showed that more topdressing ^15^N was accumulated in plants and remained in 0−200 cm soil at maturity, rather than being lost to the environment compared with the basal ^15^N in the current season. Jia et al. (2011), however, believed that basal N application contributed to lower NRE and higher residual N in soil, while excessive topdressing N inputs could increase unaccounted N losses through surface gas emissions and nitrate leaching. Ye et al. (2022) reported that N loss of basal N before jointing accounted for more than 50% of the total basal N loss during the whole growing season. In fact, roots grew slowly and mainly gathered in 0−20 cm soil layer during the seedling stage, failing to absorb the available N in deeper soil layers, which contributed to a mass loss of basal N before jointing (Ma et al., 2021). While roots and aboveground plants grew and proliferated rapidly after jointing, and had a strong demand for N. Therefore, topdressing N, which was better synchronized with the period of high N demand of plant, could be quickly absorbed or intercepted by high-density roots (Shi et al., 2012; Abubakar et al., 2022). Previous studies also indicated that large amounts of N input before sowing likely increased fertilizer-N immobilized by soil microorganisms, thus resulting in poor synchrony between fertilizer N supply and crop N demand (Sieling and Beims, 2007; Cui et al., 2010; Wang et al., 2016a). The difference in results suggested that optimizing the rate and timing of N input would be beneficial to improving N management practices.

### 4.2 The characteristics of urea-^15^N absorption and distribution in wheat under different N management strategies and preceding crops

In wheat production, the supply of fertilizer N and soil N is indispensable for the growth and development of wheat (González-Cencerrado et al., 2020). Wheat needs to absorb approximately 30 kg N ha^−1^ from soil (mainly in the form of nitrate) for each ton of grain produced (Mohammad et al., 2012). Many studies have found that the contribution of fertilizer N to crop N uptake was significantly influenced by crop yield level and soil fertility (Cao and Yin, 2015; Wang et al., 2016b). In low-yielding fields (4.7−5.4 t ha^−1^), about 45% of the total N absorbed by wheat derived from fertilizer, while the remaining 55% was from soil; While in high-yielding fields (8.0−9.0 t ha^−1^), only 15−31% of total N uptake by wheat came from fertilizer, while the larger portion (69−85%) was from soil (Wang et al., 2011). Our study showed that 39.36−44.03% of the N absorbed by wheat came from fertilizer N, and 55.97−62.60% from soil, with the yield levels of 6928.87−7257.41 kg ha^−1^ (**Tables 3**, **5** and **Supplementary Table 1**). Observations also found that the N absorbed by wheat increased by only 8.77−14.97% with the increase of N application rate by 33.33% (from 180 to 240 kg ha^−1^). The above results indicated that the contribution of fertilizer N to wheat N uptake decreased with the increase of yield level, and the high yield of wheat depended largely on the soil fertility. With the same preceding corp, higher N inputs significantly increased the total N uptake by wheat and the ratio of N derived from fertilizer in different organs, but decreased the ratio of N derived from soil. With the increasing N rate, the N uptake by wheat from the soil decreased and from urea fertilizer N significantly increased (Chen et al., 2016a). Excessive N input failed to bring high returns, but promoted the downward movement of fertilizer N, resulting in more soil N residues and losses (Wang et al., 2011). A lower N application rate in wheat cropping systems could cause more N mining from the soils, and thus less N accumulation in the soils (Jia et al., 2011).

It was observed that different wheat organs trended to absorb more N from soil (55.48−65.56%) compared with basal N (16.33−21.07%) and topdressing N (22.57−27.28%). Jia et al. (2011) also showed that with the N rate at 150−270 kg ha^−1^, N uptake in wheat derived from basal N was uniformly distributed in grain and in straw, while the N uptake derived from topdressing N in grain was 7.6–18.4% higher than in straw, which indicated that topdressing N could increase N accumulation in grain, leading to a higher ratio of grain-N to straw-N compared to basal-N application. The similar results were observed in our study, that the topdressing N absorbed by plants trended to be more allocated to grain (68.85−69.94%) compared with the basal N (63.32−68.37%). In addition, more N in grain and plant derived from topdressing N instead of basal N when the rate of basal N and topdressing N was consistent. The results demonstrated that the appropriate increase of topdressing N could not only promote the total N uptake by plants, but also regulate the distribution and transport of N to grains post-anthesis, thus promoting the N accumulation in grains (Jia et al., 2011). Fuertes-Mendizábal et al. (2018) also believed that delaying N input encouraged wheat plants to utilize N sources in a more efficient way.

### 4.3 N management strategies for improving wheat yield and N use efficiency under different preceding crops

Optimizing the timing and rates of N input is an effective way to coordinate yield component parameters and improve crop yield (Lyu et al., 2021; Zhang et al., 2023). It has been proved that N application rate and grain yield show a quadratic curve where N input increases grain yield within a certain threshold range. When N input exceeds the critical range, it decreases wheat yield, along with low N use efficiency and high environmental pollution (Yang et al., 2017; Wang et al., 2022). In our study, N240 increased N input by one-third compared with N180, but increased grain yield by only 3.49% on average in different preceding crops and years, which seems unlikely to obtain higher benefit returns (**Table 6**). With the same N inputs, the preceding maize treatment showed slightly higher yield potential compared with the preceding rice treatment, which could be due to improved soil structure or increased N mineralization rate for wheat by preceding maize (Rahimizadeh et al., 2010). Therefore, when the preceding crop is rice, it is more urgent to control N input to alleviate resource waste and environmental pollution. Abubakar et al. (2022) showed that applying 100% N to wheat either as basal or topdressing did not achieve maximum grain yield. Balancing a split fertilization program is therefore necessary as it would maintain a high grain yield and relieve the environmental burden. A study on wheat-maize rotation systems in North China Plain showed that saving N input by 60 kg ha^−1^ on the basis of conventional N practices (270 kg ha^−1^) did not decrease grain yield and wheat N uptake due to the delay of topdressing N (Liang et al., 2020). In consideration of the contribution of topdressing N to the N accumulation in wheat grains (**Table 4**), it could be inferred that split application of N fertilizer played an important role in regulating the source-sink relationship and improving yield component parameters, thereby increasing wheat yield.

Precise N management is crucial for improving plant N uptake and N efficiency (Wu et al., 2019; Zhang et al., 2023). The effect of N rates on N efficiency of winter wheat was mainly reflected in absorption efficiency and utilization efficiency. The appropriate N application rate reduced N residue in soil and enhanced N recovery efficiency, contributing to increasing wheat yield, N uptake and utilization efficiency (Wan et al., 2021a; Wang et al., 2022). However, excessive N inputs caused a significant decline in N uptake efficiency, physiological efficiency and apparent recovery fraction of applied fertilizer N (Wang et al., 2011), which could be due to the unbalanced synchronization between wheat N demand and soil N supply, ultimately resulting in considerable N loss (Li et al., 2020; Guo et al., 2021). In our study, N240 showed a reduction of 14.78−18.79% in NAE and 14.06−31.35% in NPE compared with N180, which was consistent with previous studies (**Table 6**). In addition to the N application rate, adjusting the ratio of basal N and topdressing N could also help to match the N demand by wheat with the N supply in soil, thereby improving in-season N use efficiency (Tian et al., 2018; Zhang et al., 2023). Liu et al. (2019) also indicated that optimizing N application strategies by adding topdressing N could increase grain N concentration, total N accumulation and N use efficiency. Applying topdressing N at the appropriate time and with a suitable proportion has been considered as an effective strategy for improving the synchrony among N supply, soil N availability, and crop N demand (Wang et al., 2016b; Liu et al., 2019). This study explored the difference of wheat N absorption and utilization between basal N and topdressing N, but failed to clarify the optimal ratio of basal N to topdressing N for wheat under the two preceding crops. Therefore, our further studies may focus on clarifying the appropriate proportion and timing of topdressing N for wheat under different preceding crops. The new findings will provide great help for wheat cultivation to improve fertilizer utilization and reducing environmental pollution.

## 5 Conclusion

In this study, the ^15^N tracer technique was used to analyze the fate of fertilizer N in wheat fields under different preceding crops, as well as the effect of N rate and timing on N absorption and distribution in wheat. The results suggested that the proportion of N absorbed by wheat was 39.36−44.03% from fertilizer N, and 55.97−62.60% from soil. High N inputs (240 kg ha^−1^) increased N uptake by wheat, but it obviously reduced fertilizer N recovery at the maturity stage, with an average decrease of 2.80%, and resulted in more N residue in soil and loss to the environment. In addition, with the N rate increased by 33.33% (from 180 to 240 kg ha^−1^), wheat N uptake and grain yield increased by only 8.77−14.97% and 2.51−4.49%, while NAE and NPE decreased by 14.78−18.79% and 14.06−31.35%, respectively. Observation also revealed that compared with basal N, wheat plants trended to absorb more topdressing N and allocated a higher proportion to grains (68.85−69.94%). With the same N rate, the preceding rice treatment showed higher risk of N loss into deeper soil and lower yield potential compared with the preceding maize treatment, which decreased N recovery in 0−20 cm soil, but increased 80−100 cm soil. In conclusion, reducing the N application rate and adjusting the ratio of basal N to topdressing N can effectively improve N use efficiency and alleviate environmental burden while stabilizing wheat. In the future, we will quantify all N inputs (including atmospheric deposition, straw returning and fertilizer) and outputs (including crop uptake, residue in soil, leaching loss and gaseous loss) in farmland to recommend optimal N management strategies for the different rotation systems.

## Data availability statement

The raw data supporting the conclusions of this article will be made available by the authors, without undue reservation.

## Author contributions

QM: Data curation, Investigation, Visualization, Writing – original draft. DD: Investigation, Visualization, Writing – original draft. YC: Investigation, Writing – original draft. QY: Investigation, Writing – original draft. XC: Investigation, Writing – original draft. MZ: Conceptualization, Writing – review & editing. JD: Conceptualization, Writing – review & editing. CL: Conceptualization, Writing – review & editing. WG: Conceptualization, Supervision, Writing – review & editing. GZ: Conceptualization, Supervision, Writing – review & editing. XZ: Funding acquisition, Supervision, Writing – review & editing.
