# Supplementary material for: Optimal N management affects the fate of urea-15N and improves N uptake and utilization of wheat in different rotation systems
Source: Front Plant Sci. 2024 Jul 25;15:1438215. doi: 10.3389/fpls.2024.1438215 (PMC11308437; doi:10.3389/fpls.2024.1438215)
Supplement: Supplementary file 1 [file Table_1.docx]

Supplementary material

**Table S1** Distribution of N derived from fertilizer (Ndff) and from soil (Ndfs) in wheat plants under different preceding crops and N rates in 2013−2014

| Organ | N rate | Total N uptake  (kg ha^−1^) | Ndff | | Ndfs | |
| --- | --- | --- | --- | --- | --- | --- |
|  |  |  | Amount  (kg ha^−1^) | Ratio  (%) | Amount  (kg ha^−1^) | Ratio  (%) |
| Leaf | N180 | 13.20b | 4.84b | 36.64b | 8.36a | 63.36a |
|  | N240 | 14.57a | 6.34a | 43.55a | 8.22a | 56.45b |
| Stem and sheath | N180 | 28.97b | 10.23b | 35.32b | 18.74b | 64.68a |
|  | N240 | 37.69a | 16.53a | 43.85a | 21.16a | 56.15b |
| Grain | N180 | 123.53b | 52.67b | 42.64a | 70.86b | 57.36a |
|  | N240 | 138.10a | 61.27a | 44.36a | 76.83a | 55.64b |
| Ear axis and glume | N180 | 14.16b | 5.07b | 35.79b | 9.09a | 64.21a |
|  | N240 | 16.03a | 6.74a | 42.06a | 9.29a | 57.94b |
| Total | N180 | 179.86b | 72.81b | 40.48b | 107.05b | 59.52a |
|  | N240 | 206.39a | 90.88a | 44.03a | 115.51a | 55.97b |

**Table S2** Variance analyses for the effects of Preceding crop and N rate on total N uptake, N derived from fertilizer (Ndff) and soil (Ndfs) in different organs of wheat in 2014−2015

| Ogran | Factor | Total N uptake | Ndff | | Ndfs | |
| --- | --- | --- | --- | --- | --- | --- |
|  |  |  | Amount | Ratio | Amount | Ratio |
| Leaf | Preceding crop (P) | 10.52^*^ | 18.63^*^ | 1.77^ns^ | 6.46^ns^ | 0.73^ns^ |
|  | N rate (N) | 101.32^**^ | 404.05^**^ | 106.67^**^ | 12.36^*^ | 43.76^**^ |
|  | P × N | 2.51^ns^ | 4.70^ns^ | 0.91^ns^ | 1.45^ns^ | 0.37^ns^ |
| Stem and sheath | Preceding crop (P) | 16.35^*^ | 12.06^*^ | 0.06^ns^ | 19.30^*^ | 19.30^*^ |
|  | N rate (N) | 208.58^**^ | 682.11^**^ | 153.53^**^ | 46.36^**^ | 46.36^**^ |
|  | P × N | 0.28^ns^ | 4.06^ns^ | 3.57^ns^ | 0.19^ns^ | 0.18^ns^ |
| Grain | Preceding crop (P) | 2.99^ns^ | 24.06^**^ | 10.10^*^ | 8.32^*^ | 5.16^ns^ |
|  | N rate (N) | 17.13^*^ | 121.16^**^ | 47.82^**^ | 9.68^*^ | 24.45^**^ |
|  | P × N | 1.51^ns^ | 0.06^ns^ | 1.34^ns^ | 3.71^ns^ | 0.68^ns^ |
| Ear axis and glume | Preceding crop (P) | 37.59^**^ | 61.85^**^ | 3.40^ns^ | 24.64^**^ | 1.47^ns^ |
|  | N rate (N) | 7.45^*^ | 132.83^**^ | 83.65^**^ | 11.84^*^ | 36.26^**^ |
|  | P × N | 0.08^ns^ | 1.56^ns^ | 2.08^ns^ | 0.13^ns^ | 0.90^ns^ |
| Total | Preceding crop (P) | 2.85^ns^ | 14.17^*^ | 4.49^ns^ | 0.06^ns^ | 2.14^ns^ |
|  | N rate (N) | 38.89^**^ | 201.23^**^ | 64.92^*^ | 5.45^*^ | 30.98^**^ |
|  | P × N | 0.42^ns^ | 0.05^ns^ | 1.16^ns^ | 1.58^ns^ | 0.55^ns^ |

**Table S3** Distribution of N derived from basal fertilizer (Ndfbf) and from topdressing fertilizer (Ndftf) in wheat plants under N rate of 240 kg ha^−1^ in 2013−2014

| Organ | Ndfbf | | Ndftf | |
| --- | --- | --- | --- | --- |
|  | Amount  (kg ha^−1^) | Ratio  (%) | Amount  (kg ha^−1^) | Ratio  (%) |
| Leaf | 3.04 | 20.89 | 3.36 | 23.05 |
| Stem and sheath | 7.94 | 21.07 | 8.51 | 22.57 |
| Grain | 23.38 | 16.93 | 37.41 | 27.09 |
| Ear axis and glume | 2.62 | 16.33 | 4.22 | 26.30 |
| Total | 36.99 | 17.92 | 53.49 | 25.92 |
